# Supplementary material for: The chromatin landscape of high-grade serous ovarian cancer metastasis identifies regulatory drivers in post-chemotherapy residual tumour cells
Source: Commun Biol. 2024 Sep 28;7:1211. doi: 10.1038/s42003-024-06909-9 (PMC11438996; doi:10.1038/s42003-024-06909-9)
Supplement: Supplementary file 2 — Supplementary Information [file 42003_2024_6909_MOESM2_ESM.pdf]

Supplementary Information

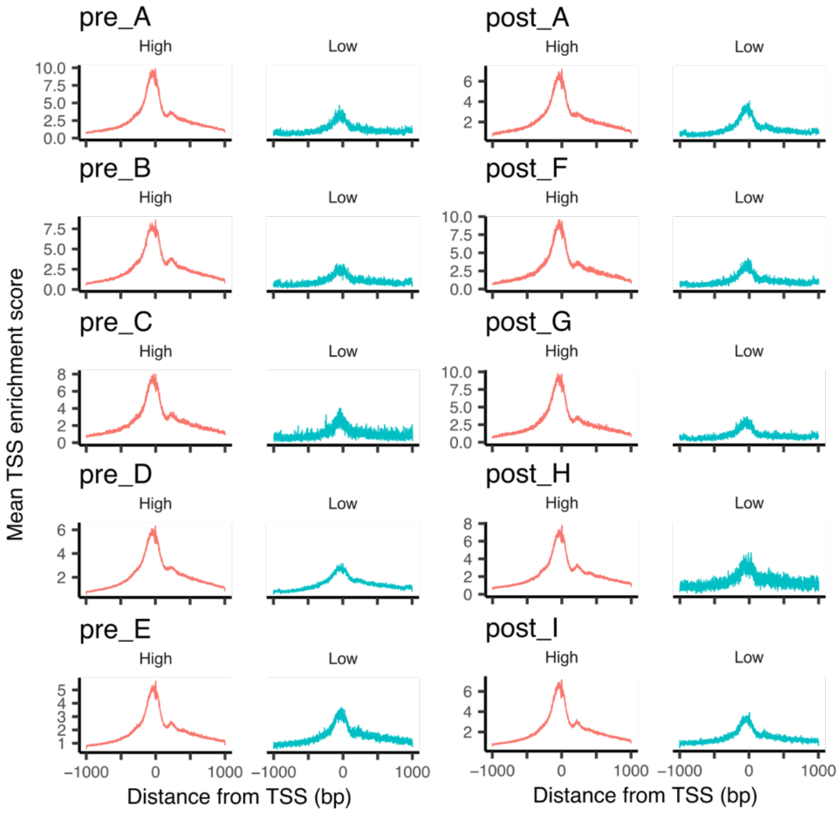

Supplementary Figure S1. Per-sample transcription start site enrichment density profiles. Average read density profiles centered on transcription start sites +/1 1000bp.

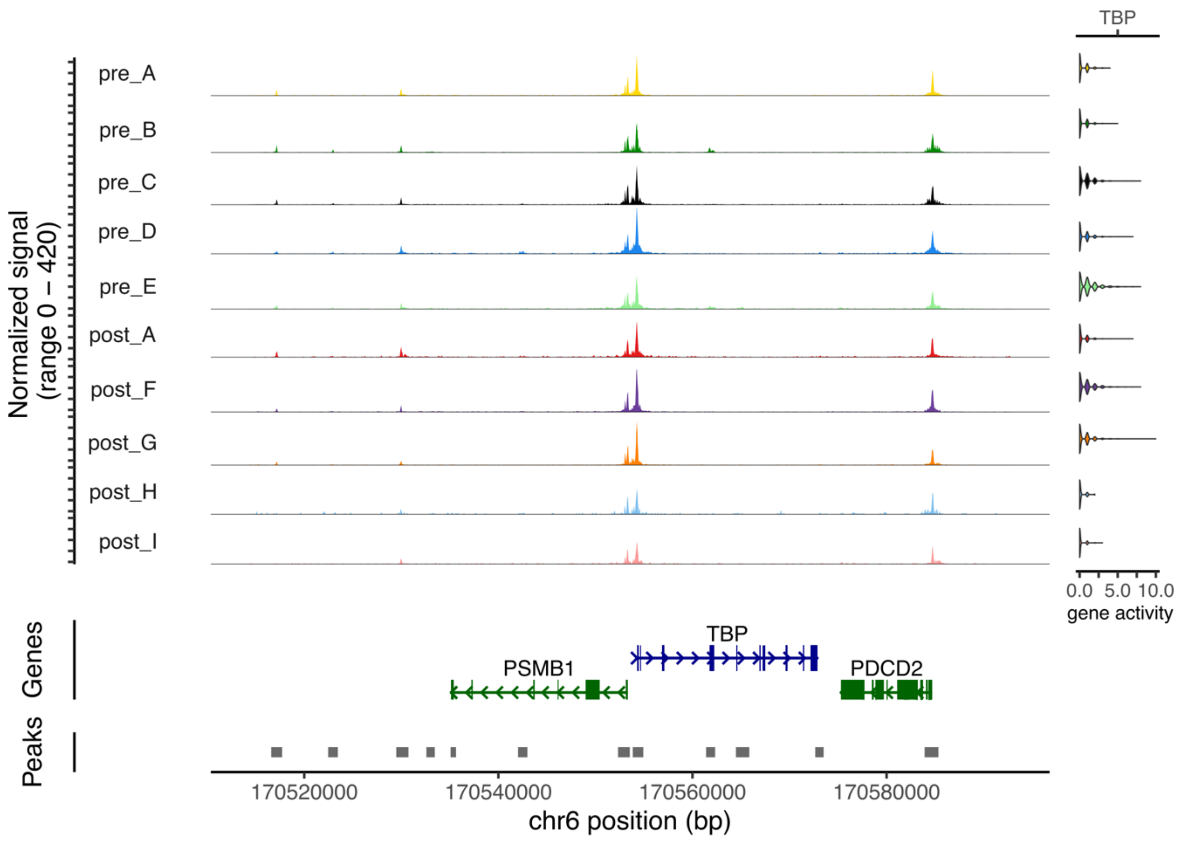

Supplementary Figure S2. Per-sample read density profile at Tata Binding Protein (TBP) genomic loci. Aggregated read coverage profiles at a ubiquitously open chromatin loci.

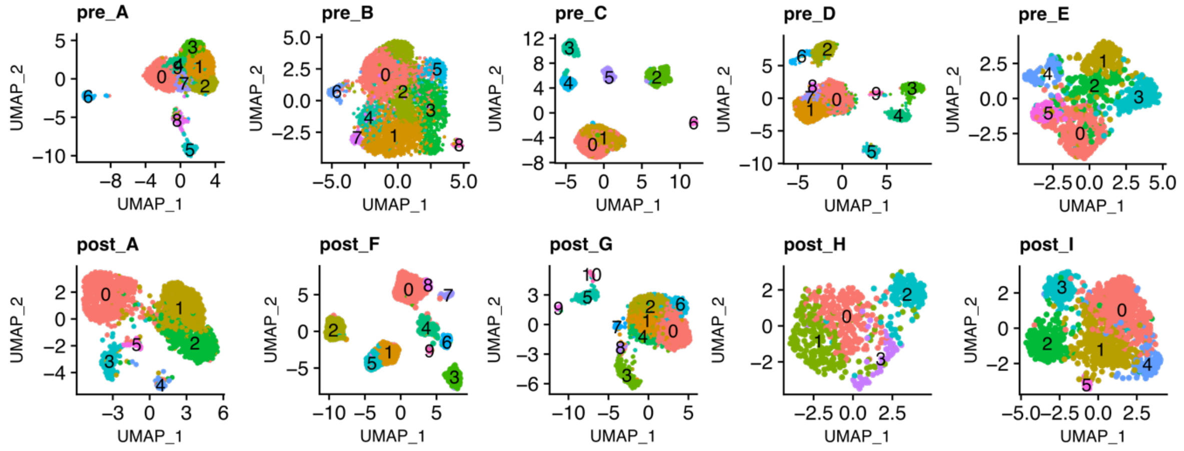

Supplementary Figure S3. Per-sample unsupervised clustering. UMAP embedding of scATAC-seq peak data for each sample overlaid with unsupervised clustering labels.

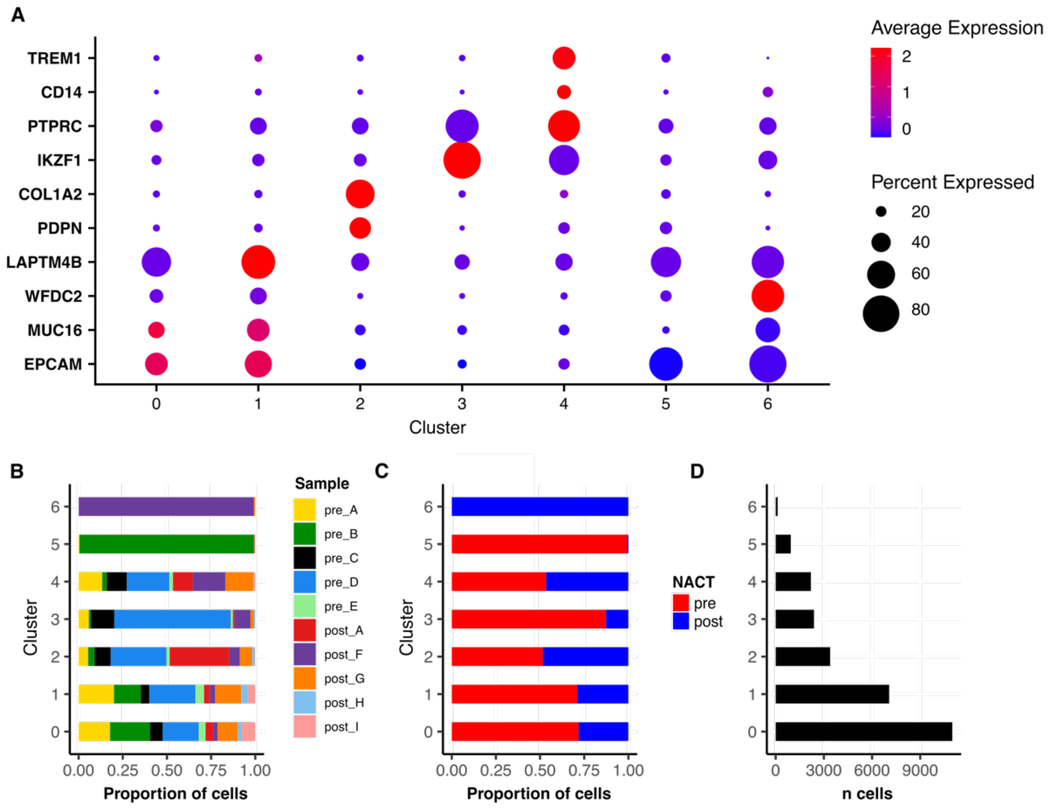

Supplementary Figure S4. Integrated data unsupervised clustering profile. A Average cluster-wise gene activity scores of major cell lineage defining genes. Dot size indicates percentage of the cluster with gene score indicating expression. Sample composition (**B**), NACT treatment composition (**C**) and total number of cells (**D**) for each cluster.

Supplementary Table S1. Per sample major lineage cell counts

Sample	Epithelial	Fibroblast	Lymphocyte	Myeloid	Total
pre_A	3365	180	137	291	3973
pre_B	4503	128	27	58	4716
pre_C	1119	306	316	248	1989
pre_D	4082	1072	1573	527	7254
pre_E	780	63	28	45	916
post_A	698	1140	21	246	2105
post_F	618	205	215	405	1443
post_G	2299	238	47	347	2931
post_H	493	50	19	6	568
post_I	1165	10	1	18	1194

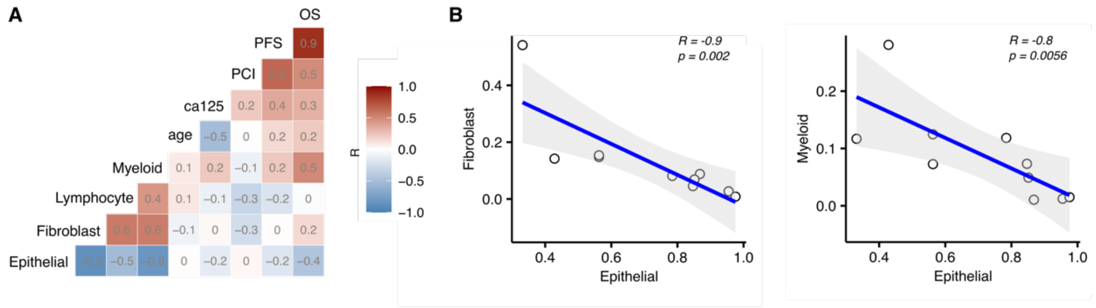

Supplementary Figure S5. Major lineage cell type correlations. A Spearman correlation matrix of major cell type proportions in all patients (n=10) with clinical covariates. **B** Scatter plots showing inverse correlation of Epithelial with Fibroblast and Myeloid cell type proportions.

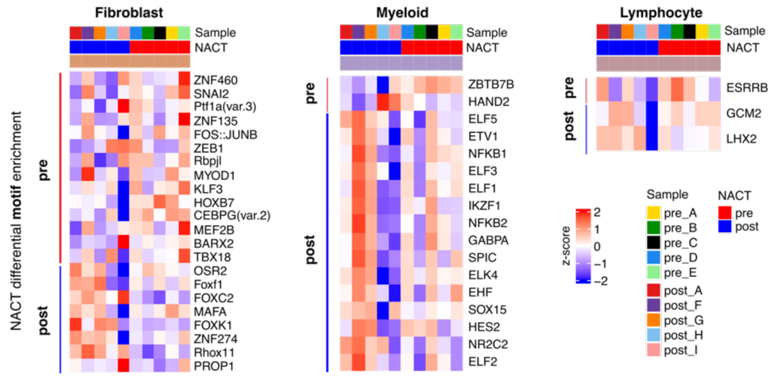

Supplementary Figure S6. Modulation of transcription factor binding motif enrichments in major lineage cell types following chemotherapy. Within patient-cell-type average transcription factor motif chromVar activity score profile of motifs identified as post vs pre chemotherapy differentially enriched (adjusted p < 0.01) in Fibroblast Myeloid and Lymphocyte cells.

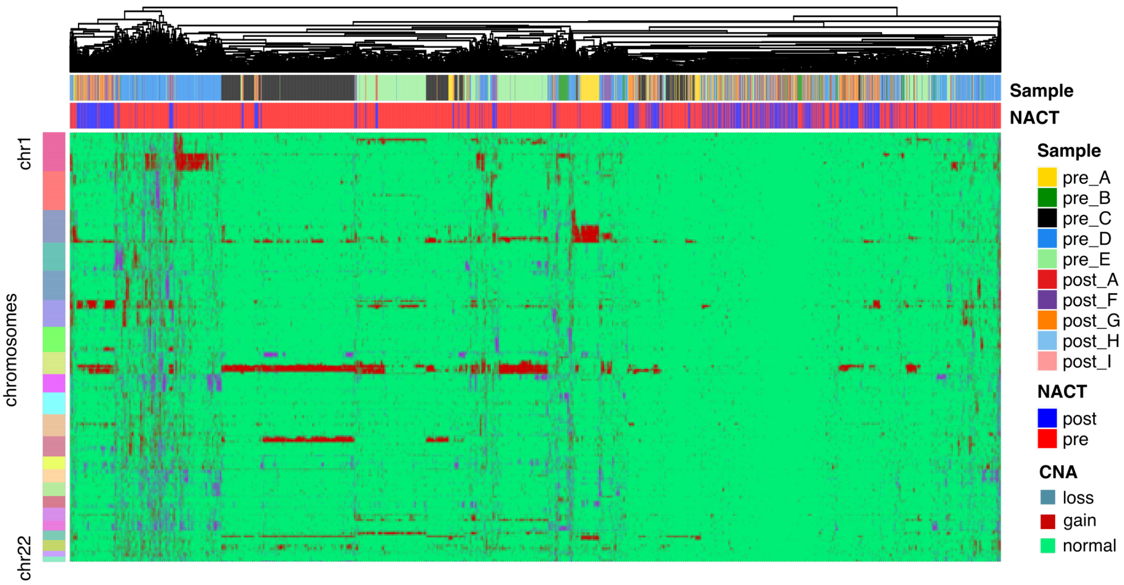

Supplementary Figure S7. Epithelial tumor cell copy number alterations. Hierarchical clustering of copy number alteration profile in epithelial tumor cells. CNAs were calculated with EpiAneufinder.

Supplementary Table S2. Stress-associated chemoresistant tumor cell signature genes

Gene name	Description	Location
**CEBPB**	CCAAT enhancer binding protein beta	20q13.13
**CEBPD**	CCAAT enhancer binding protein delta	8q11.21
**FOS**	Fos proto-oncogene, AP-1 transcription factor subunit	14q24.3
**IL6**	interleukin 6	7p15.3
**JUN**	Jun proto-oncogene, AP-1 transcription factor subunit	1p32.1
**JUNB**	JunB proto-oncogene, AP-1 transcription factor subunit	19p13.13
**MCL1**	MCL1 apoptosis regulator, BCL2 family member	1q21.2
**MYC**	MYC proto-oncogene, bHLH transcription factor	8q24.21
**SOCS3**	suppressor of cytokine signaling 3	17q25.3
**ATF3**	activating transcription factor 3	1q32.3
**DUSP1**	dual specificity phosphatase 1	5q35.1
**EGR1**	early growth response 1	5q31.2
**FOSB**	FosB proto-oncogene, AP-1 transcription factor subunit	19q13.32
**CEBPA**	CCAAT enhancer binding protein alpha	19q13.11
**DDIT3**	DNA damage inducible transcript 3	12q13.3
**EGR2**	early growth response 2	10q21.3
**CDKN1A**	cyclin dependent kinase inhibitor 1A	6p21.2
**GADD45B**	growth arrest and DNA damage inducible beta	19p13.3
**TNF**	tumor necrosis factor	6p21.33
**HES1**	hes family bHLH transcription factor 1	3q29

**HBEGF**	heparin binding EGF like growth factor	5q31.3
**BCL6**	BCL6 transcription repressor	3q27.3
**NR4A1**	nuclear receptor subfamily 4 group A member 1	12q13.13
**DUSP6**	dual specificity phosphatase 6	12q21.33
**GADD45G**	growth arrest and DNA damage inducible gamma	9q22.2
**ID2**	inhibitor of DNA binding 2	2p25.1
**NFKBIA**	NFKB inhibitor alpha	14q13.2
**PLK3**	polo like kinase 3	1p34.1
**SNAI2**	snail family transcriptional repressor 2	8q11.21
**CREB5**	cAMP responsive element binding protein 5	7p15.1-p14.3
**HLA-G**	major histocompatibility complex, class I, G	6p22.1
**HIST1H2BC**	H2B clustered histone 4	6p22.2
**CALML3**	calmodulin like 3	10p15.1
**SNAI1**	snail family transcriptional repressor 1	20q13.13

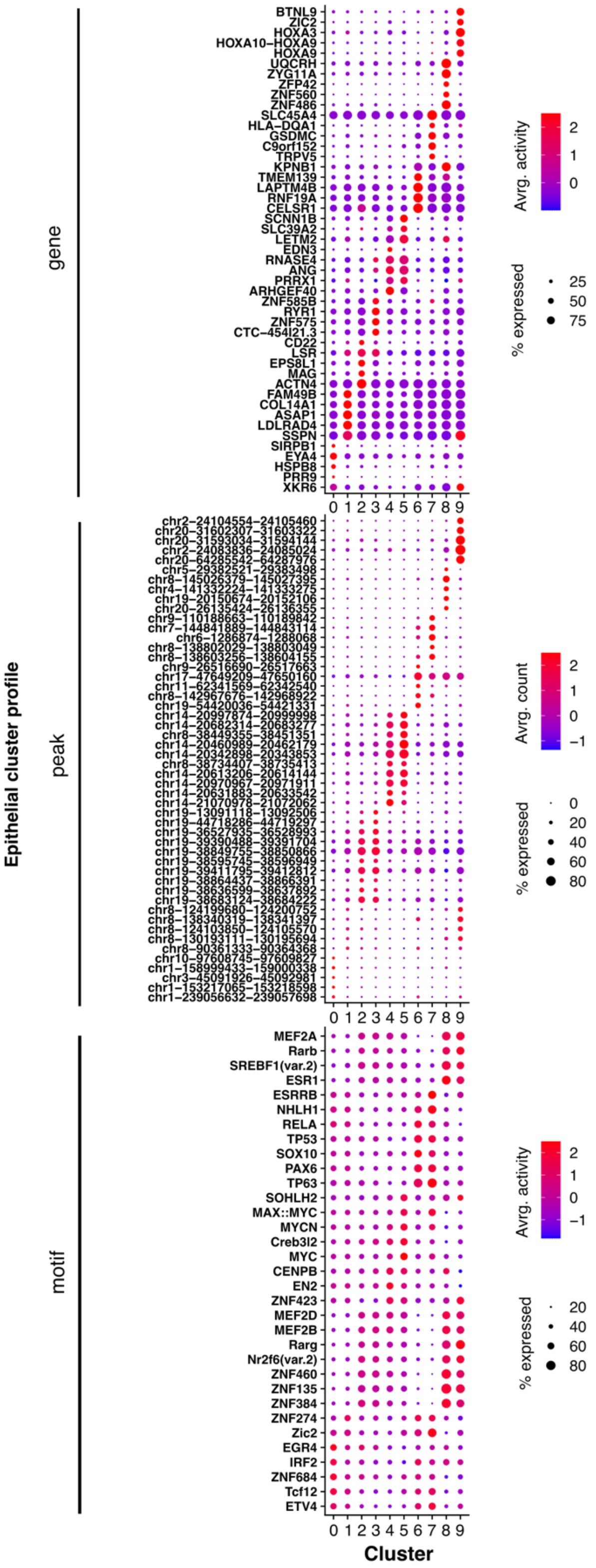

Supplementary Figure S8. Profile of HGSOC tumour cell subpopulations. Scaled average gene activity (top), counts at peak region (middle) and motif activity score (bottom) of the top 5 marker features for each epithelial tumour cell subclusters identified by unsupervised clustering.

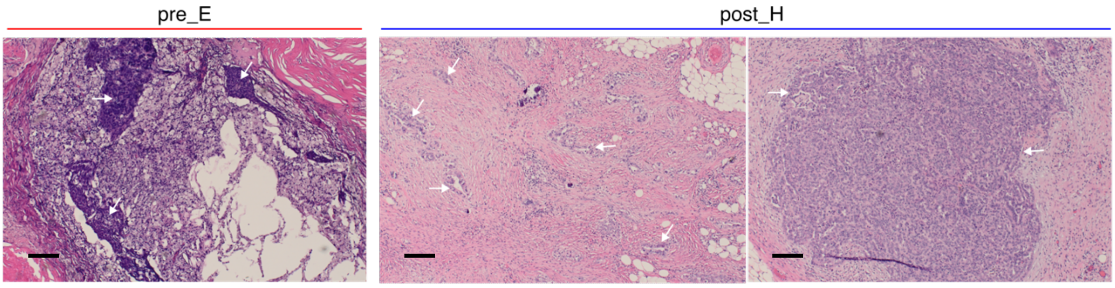

Supplementary Figure S9. High grade serous carcinoma in omental resection from pre neoadjuvant chemotherapy (pre_E) and two regions from post neoadjuvant chemotherapy tissue samples (formalin fixed, paraffin embedded and haematoxylin & eosin stained surgical pathology specimens; scale bars 100um).

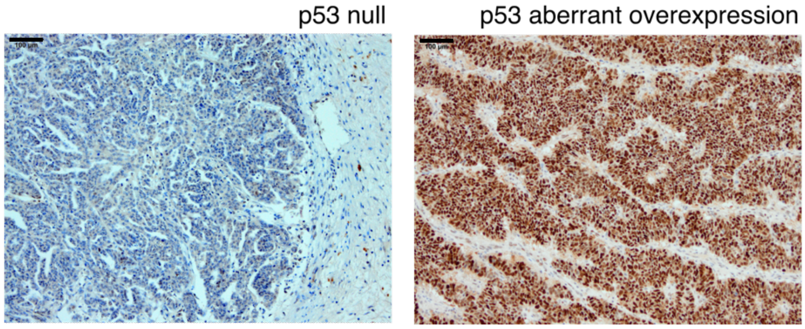

Supplementary Figure S10. p53 Immunohistochemistry staining in high grade serous ovarian carcinoma omental resection. Representative images of HGSOC omental tissue sections stained for p53 showing p53 null phenotype and aberrant overexpression. Scale bars 100um.

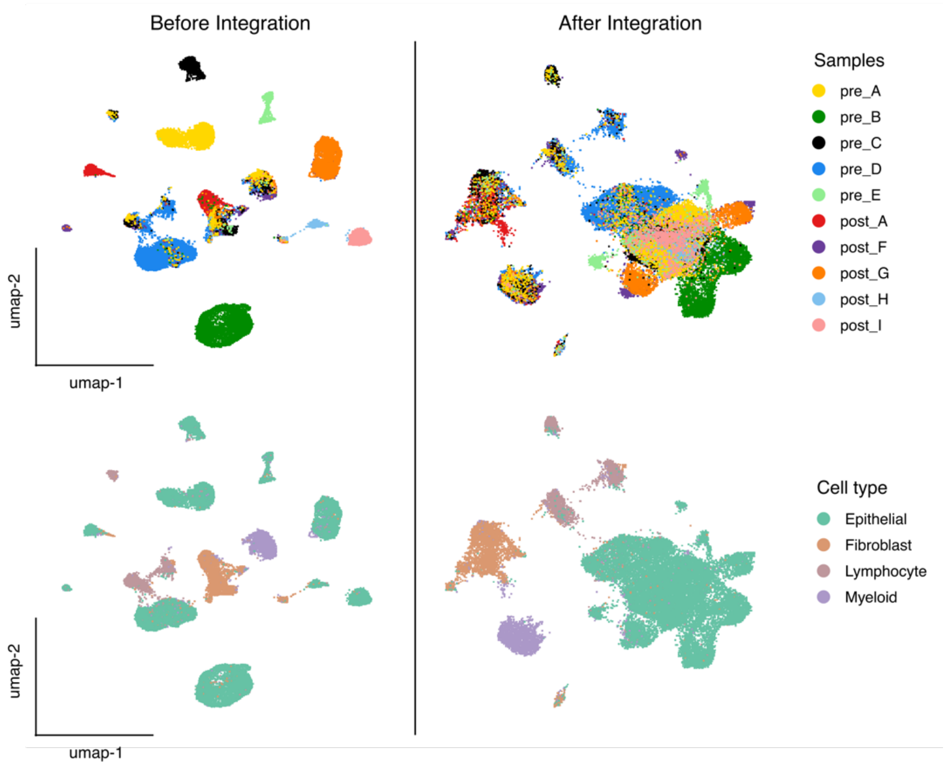

Supplementary Figure S11. Harmony integration. UMAP embedding of merged (Before Integration) and Harmony-integrated (After Integration) scATAC-seq data overlaid with patient sample label (top) and major lineage cell type (bottom).
